# Sweetened Beverage Intake and Incident Chronic Kidney Disease in the UK Biobank Study

**DOI:** 10.1001/jamanetworkopen.2023.56885

**Published:** 2024-02-28

**Authors:** Ga Young Heo, Hee Byung Koh, Jung Tak Park, Seung Hyeok Han, Tae-Hyun Yoo, Shin-Wook Kang, Hyung Woo Kim

**Affiliations:** 1 Department of Internal Medicine, Institute of Kidney Disease Research, Yonsei University College of Medicine, Seoul, Republic of Korea; 2 Division of Nephrology, International Saint Mary’s Hospital, Catholic Kwandong University, Incheon, Republic of Korea; 3 Institute for Innovation in Digital Healthcare, Yonsei University, Seoul, Republic of Korea

## Abstract

**Importance:**

An increasing body of evidence indicates an association between consuming sugar or its alternatives and cardiometabolic diseases. However, the effects of the consumption of sugar-sweetened beverages, artificially sweetened beverages, and natural juices on kidney health remain unclear.

**Objective:**

To investigate the association of the intake of sugar-sweetened beverages, artificially sweetened beverages, and natural juices with the risk of chronic kidney disease (CKD), and the effect of substituting these beverage types for one another on this association.

**Design, Setting, and Participants:**

This prospective, population-based cohort study analyzed data from the UK Biobank. Participants without a history of CKD who completed at least 1 dietary questionnaire were included. The follow-up period was from the date of the last dietary questionnaire until October 31, 2022, in England; July 31, 2021, in Scotland; and February 28, 2018, in Wales. Data were analyzed from May 1 to August 1, 2023.

**Exposures:**

Consumption of sugar-sweetened beverages, artificially sweetened beverages, and natural juices.

**Main Outcomes and Measures:**

The primary outcome was incident CKD. Multivariable Cox proportional hazards models were used to estimate the associations between the 3 beverage types and incident CKD. A substitution analysis was used to evaluate the effect on the associations of substituting one beverage type for another.

**Results:**

A total of 127 830 participants (mean [SD] age, 55.2 [8.0] years; 66 180 female [51.8%]) were included in the primary analysis. During a median (IQR) follow-up of 10.5 (10.4-11.2) years, 4459 (3.5%) cases of incident CKD occurred. The consumption of more than 1 serving per day of sugar-sweetened beverages was associated with higher risk of incident CKD (adjusted hazard ratio [AHR], 1.19 [95% CI, 1.05-1.34]) compared with not consuming sugar-sweetened beverages. The AHR for participants consuming more than 0 to 1 serving per day of artificially sweetened beverages was 1.10 (95% CI, 1.01-1.20) and for consuming more than 1 serving per day was 1.26 (95% CI, 1.12-1.43) compared with consuming no artificially sweetened beverages. By contrast, there was no significant association between natural juice intake and incident CKD (eg, for >1 serving per day: HR, 0.99 [95% CI, 0.87-1.11]; *P* = .10). Substituting sugar-sweetened beverages with artificially sweetened beverages did not show any significant difference in the risk of CKD (HR, 1.03 [95% CI, 0.96-1.10]). Conversely, replacing 1 serving per day of sugar-sweetened beverage with natural juice (HR, 0.93 [95% CI, 0.87-0.97]) or water (HR, 0.93 [95% CI, 0.88-0.99]) or replacing 1 serving per day of artificially sweetened beverage with natural juice (HR, 0.90 [95% CI, 0.84-0.96]) or water (HR, 0.91 [95% CI, 0.86-0.96]) was associated with a reduced risk of incident CKD.

**Conclusions and Relevance:**

Findings from this cohort study suggest that lower consumption of sugar-sweetened beverages or artificially sweetened beverages may reduce the risk of developing CKD.

## Key Points

**Question:**

Is there an association between the consumption of sweetened beverages and kidney health?

**Findings:**

In this cohort study of 127 830 adults in UK Biobank data, consuming more than 1 serving per day of sugar-sweetened beverages or any artificially sweetened beverages was associated with increased risk of incident chronic kidney disease. Substituting 1 serving per day of sugar-sweetened beverages or artificially sweetened beverages with natural juices or water was associated with reduced risk of incident chronic kidney disease.

**Meaning:**

These findings suggest that healthy beverage consumption habits may help prevent the development of chronic kidney disease.

## Teaser

This cohort study assesses whether the consumption of sugar-sweetened, artificially sweetened, or natural juice beverages is associated with the risk of developing chronic kidney disease and whether substituting one of these beverage types for another alters that association among adults.

## Introduction

Chronic kidney disease (CKD) is a significant global health problem, affecting more than 800 million people worldwide.^1^ Given the irreversible nature of CKD, lifestyle modification should be encouraged to prevent it.^2^ Among these factors, dietary modification is the cornerstone to preventing and managing CKD.^3^ Beverage intake is an important part of dietary intake due to its potential to influence on fluid balance, nutrient intake, and metabolic pathways^4^; however, the association between beverage consumption and CKD risk is not well established.

An increasing body of evidence indicates that there is an association between consuming sweetened beverages and cardiometabolic diseases.^5,6^ To address this concern, the World Health Organization recommended limiting free sugar intake to less than 5% to 10% of total energy intake.^7^ Interestingly, while artificially sweetened beverages are usually considered alternatives to sugar-sweetened beverages, previous studies have reported that artificial sweeteners may be associated with an increased risk of metabolic disorders, such as type 2 diabetes, cardiovascular diseases, and mortality.^5,8^ In alignment with those results, the World Health Organization guideline suggests that artificial sweeteners should not be used to achieve weight control or reduce the risk of noncommunicable diseases.^9^ Even natural juices, which contain natural nutrients, should be consumed with caution, as they have been associated with weight gain, the development of metabolic syndrome, and type 2 diabetes.^10,11^

Although sugar-sweetened beverages, artificially sweetened beverages, and natural juices are associated with adverse health conditions that are closely related to kidney health, limited mention of CKD prevention is found in the current guidelines.^7,9^ Previous studies on the association between sweetened beverage consumption and kidney health are limited and show conflicting results.^12,13,14,15,16^ Given this background, the present study used the UK Biobank cohort to investigate the association between the intake of 3 types of beverage (sugar-sweetened beverages, artificially sweetened beverages, and natural juices) and the risk of incident CKD, and the effect of substituting beverage types on this association.

## Methods

### Study Population and Data Collection

The UK Biobank is a prospective population-based cohort study that has recruited over 500 000 participants 40 to 69 years of age between 2006 and 2010.^17^ Detailed data collection and measurement methods are described in eMethods 1 in Supplement 1. The follow-up period of the present study was from the date of the last dietary questionnaire until October 31, 2022, in England; July 31, 2021, in Scotland; and February 28, 2018, in Wales. UK Biobank study approval was obtained from the National Health Service and the National Research Ethics Service and was renewed. All participants provided written informed consent, and the records for the withdrawn participants were removed. This study followed the Strengthening the Reporting of Observational Studies in Epidemiology (STROBE) reporting guideline.

**Figure 1.**
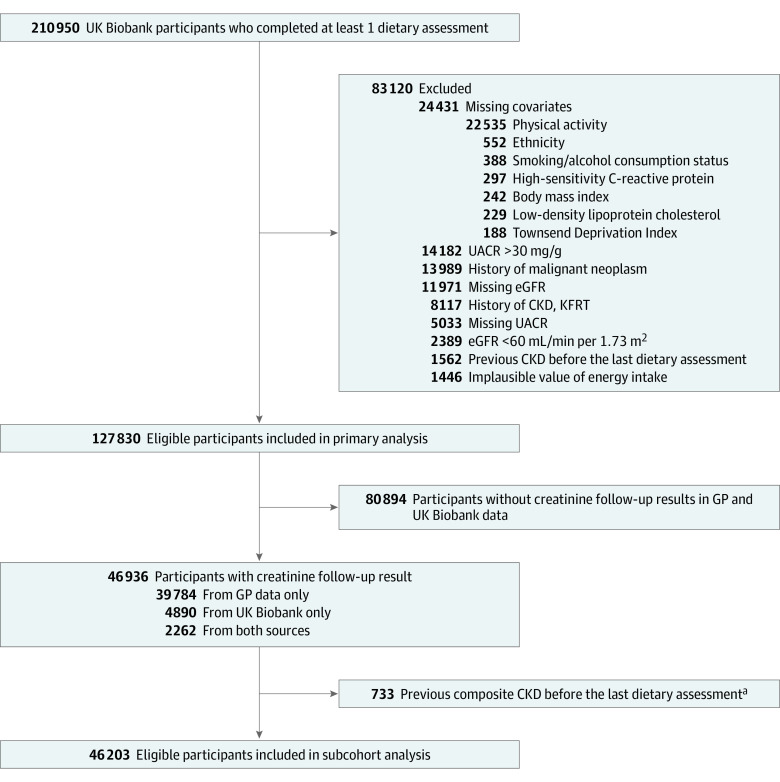
Study Participant Flow CKD indicates chronic kidney disease; eGFR, estimated glomerular filtration rate; GP, general practice; KFRT, kidney failure with replacement therapy; and UACR, urinary albumin to creatinine ratio. ^a^Composite CKD outcome was defined based on diagnosis codes or measurements of eGFR below 60 mL/min/1.73 m^2^, whichever came first.

This study initially screened 210 950 participants enrolled from 2006 to 2010, who completed at least 1 online dietary questionnaire in the UK Biobank (Figure 1). Race and ethnicity categories in UK Biobank included Asian; Black; White; and Multiethnic or other, which included Black and White Caribbean, Black and White African, Asian and White, other mixed background, mixed, or other ethnic group. Race and ethnicity were assessed in the present study to adjust for the potential disparities of race and ethnicity in CKD. To investigate the risk of developing CKD, participants with missing data for estimated glomerular filtration rate or urinary albumin to creatinine ratio, an estimated glomerular filtration rate lower than 60 mL/min/1.73 m^2^, a urinary albumin to creatinine ratio higher than 30 mg/g or a history of CKD or kidney failure with replacement therapy at baseline were excluded. This study also excluded participants who developed CKD before the last dietary questionnaire was completed. Participants who had a history of malignant neoplasm, missing data for covariates, or implausible values for energy intake (defined as energy intake of <500 or >3500 kcal/d in women, and <800 or >4000 kcal/d in men)^18^ were also excluded. Furthermore, we constructed a subcohort data set that included participants with at least 1 follow-up creatinine measurement obtained from either UK Biobank or general practice data. Participants with a composite event before the last dietary questionnaire was completed were excluded from the sensitivity analysis (Figure 1).

### Exposure Assessment

Daily beverage intake was assessed by following question: “How many glasses, cans, or cartons containing 250 mL of sugar-sweetened beverages, artificially sweetened beverages, or natural juices did you drink yesterday?” Participants were categorized into 3 groups based on the consumption of these products: 0, more than 0 to 1, and more than 1 serving per day. Dietary information was collected up to 5 times. The mean dietary intake was used as a primary exposure to estimate the usual consumption.

### Outcome Measures

The primary outcome of the study was incident CKD, defined using *International Statistical Classification of Diseases, Tenth Revision* codes, Office of Population, Censuses and Survey’s Classification of Interventions and Procedures codes, clinical codes^19^ in hospital inpatient data, death register records, and primary care data (eMethods 2 and eTable 2 in Supplement 1). In the subcohort data set, the composite CKD outcome was based on diagnostic codes or estimated glomerular filtration rate lower than 60 mL/min/1.73 m^2^, whichever came first. The outcomes were assessed from the date of the last dietary questionnaire completed until the date of CKD development, death, or the last follow-up, whichever came first.

### Statistical Analysis

Baseline characteristics were examined according to the 3 types of beverages. Continuous variables and categorical variables were expressed as means (SDs) and numbers (percentage), respectively. The primary analysis used a Cox proportional hazards model to estimate the association between 3 types of beverages and incident CKD. The results are presented as adjusted hazard ratios (AHRs) and 95% CIs. Detailed models are described in eMethods 3 in Supplement 1. An adjusted survival curve for the incidence of CKD was calculated using estimated survival from the fully adjusted Cox model with the same covariates as in the primary analysis. We conducted a substitution analysis to evaluate the effect of substituting 1 beverage for another.^20^ We also performed a mediation analysis to quantify the proportion of CKD risk explained by indirect factors, including sugar intake and BMI, as well as the direct association of sugar-sweetened beverage intake and artificially sweetened beverage intake.^21^ Detailed methods for substitution and mediation analyses are described in eMethods 4 in Supplement 1. To assess the robustness of our findings, several sensitivity analyses were conducted. First, we used the dietary information from the first completed dietary questionnaire instead of the mean value. Second, we excluded participants who developed CKD events in first 3 years of follow-up to address potential reverse association. Third, the analysis was repeated among participants who completed more than 2 dietary assessments. Fourth, we adjusted the household income, rather than the Townsend Deprivation Index score. Fifth, to deal with missing baseline covariates, we used imputation methods such as single imputation, missing indicator method, and multiple imputation (eMethod 5 in Supplement 1). Sixth, we analyzed the association between the intake of 3 types of beverage and the composite CKD outcome in the subcohort. In addition, we examined the subgroup analysis detailed in eMethods 6 in Supplement 1. We considered statistical significance as a 2-sided *P* < .05, and all analyses were performed from May 1 to August 1, 2023 using Stata, version 17.0 (StataCorp).

## Results

### Baseline Characteristics of the Study Population

The baseline characteristics of the study population according to the consumption of the different beverages are shown in Table 1. A total of 127 830 participants (mean [SD] age, 55.2 [8.0] years; 66 180 [51.8%] female, and 61 650 [48.2%] male) were included in the primary analysis. Among the participants, 41 427 consumed sugar-sweetened beverages, 26 295 consumed artificially sweetened beverages, and 66 658 consumed natural juices. Among participants who completed the dietary questionnaires 5 times (median, 2.0 [IQR, 1.0-3.0]) (eTable 1 in Supplement 1), the Pearson correlation coefficients between beverage intake from the first assessment and the mean value were 0.70 for sugar-sweetened beverages, 0.73 for artificially sweetened beverages, and 0.77 for natural juices. Participants with higher intake of sugar-sweetened beverages more often were younger and male, had a higher body mass index (BMI, calculated as weight in kilograms divided by height in meters squared), and consumed more total energy and sugar compared with participants with lower sugar-sweetened beverage intake. Moreover, they had higher levels of triglycerides and high-sensitivity C-reactive protein (hsCRP). Participants with a higher consumption of artificially sweetened beverages more often were younger and female and had a higher BMI compared with participants with a lower consumption of artificially sweetened beverages. Compared with participants who did not consume artificially sweetened beverages, participants with higher consumption of artificially sweetened beverages showed a slightly increased energy intake and higher levels of triglycerides and hsCRP. However, there was no significant difference between artificially sweetened beverage consumption and sugar intake. Participants consuming natural juices had higher total energy and sugar intake, were more often male, and had relatively higher triglyceride levels than participants with lower intakes of natural juices.

**Table 1.** Baseline Characteristics of Participants by Category of Beverage Intake

Characteristics^a^	Participants, No. (%)								
	Sugar-sweetened beverages			Artificially sweetened beverages			Natural juices		
	0/d (n = 86 403)	>0-1/d (n = 33 028)	>1/d (n = 8399)	0/d (n = 101 535)	>0-1/d (n = 19 446)	>1/d (n = 6849)	0/d (n = 61 172)	>0-1/d (n = 57 384)	>1/d (n = 9274)
Age, mean (SD), y	55.6 (7.9)	54.9 (8.1)	52.7 (8.0)	55.7 (7.9)	54.0 (7.9)	52.4 (8.0)	54.9 (8.0)	55.6 (7.9)	54.9 (8.0)
Sex									
Female	46 157 (53.4)	16 509 (50.0)	3514 (41.8)	51 442 (50.7)	10 870 (55.9)	3868 (56.5)	33 282 (54.4)	28 778 (50.1)	4120 (44.4)
Male	40 246 (46.6)	16 519 (50.0)	4885 (58.2)	50 093 (49.3)	8576 (44.1)	2981 (43.5)	27 890 (45.6)	28 606 (49.9)	5154 (55.6)
Race and ethnicity									
Asian	1355 (1.6)	576 (1.7)	124 (1.5)	1696 (1.7)	287 (1.5)	72 (1.1)	1160 (1.9)	774 (1.3)	121 (1.3)
Black	755 (0.9)	457 (1.4)	235 (2.8)	1156 (1.1)	222 (1.1)	69 (1.0)	719 (1.2)	506 (0.9)	222 (2.4)
White	83 225 (96.3)	31 493 (95.4)	7898 (94.0)	97 314 (95.8)	18 685 (96.1)	6617 (96.6)	58 431 (95.5)	55 452 (96.6)	8733 (94.2)
Multiethnic or other^b^	1068 (1.2)	502 (1.5)	142 (1.7)	1 369 (1.3)	252 (1.3)	91 (1.3)	862 (1.4)	652 (1.1)	198 (2.1)
Townsend Deprivation Index	−1.6 (2.8)	−1.6 (2.8)	−1.4 (3.0)	−1.6 (2.9)	−1.7 (2.8)	−1.5 (3.0)	−1.5 (2.9)	−1.8 (2.8)	−1.4 (3.0)
Household income, €/y^c^									
<18 000	10 686 (12.4)	4002 (12.1)	1167 (13.9)	12 846 (12.7)	2202 (11.3)	807 (11.8)	8657 (14.2)	6155 (10.7)	1043 (11.2)
18 000-30 999	17 880 (20.7)	6928 (21.0)	1759 (20.9)	21 298 (21.0)	3921 (20.2)	1348 (19.7)	13 198 (21.6)	11 646 (20.3)	1723 (18.6)
31 000-51 999	22 713 (26.3)	8892 (26.9)	2307 (27.5)	26 938 (26.5)	5099 (26.2)	1875 (27.4)	16 151 (26.4)	15 448 (26.9)	2313 (24.9)
52 000-99 999	21 124 (24.4)	8165 (24.7)	1955 (23.3)	24 393 (24.0)	5043 (25.9)	1808 (26.4)	13 912 (22.7)	14 785 (25.8)	2547 (27.5)
≥100 000	6806 (7.9)	2340 (7.1)	554 (6.6)	7596 (7.5)	1589 (8.2)	515 (7.5)	3931 (6.4)	4810 (8.4)	959 (10.3)
Missing value	7194 (8.3)	2701 (8.2)	657 (7.8)	8464 (8.3)	1592 (8.2)	496 (7.2)	5323 (8.7)	4540 (7.9)	689 (7.4)
Alcohol consumption									
Never	2235 (2.6)	1081 (3.3)	343 (4.1)	2838 (2.8)	571 (2.9)	250 (3.7)	1924 (3.1)	1399 (2.4)	336 (3.6)
Previous	2197 (2.5)	949 (2.9)	392 (4.7)	2661 (2.6)	546 (2.8)	331 (4.8)	1961 (3.2)	1295 (2.3)	282 (3.0)
Current	81 971 (94.9)	30 998 (93.9)	7664 (91.2)	96 036 (94.6)	18 329 (94.3)	6268 (91.5)	57 287 (93.6)	54 690 (95.3)	8656 (93.3)
Smoking status									
Never	48 756 (56.4)	19 506 (59.1)	4899 (58.3)	58 315 (57.4)	11 117 (57.2)	3729 (54.4)	33 330 (54.5)	34 139 (59.5)	5692 (61.4)
Previous	30 835 (35.7)	11 070 (33.5)	2657 (31.6)	35 169 (34.6)	6901 (35.5)	2492 (36.4)	22 081 (36.1)	19 590 (34.1)	2891 (31.2)
Current	6812 (7.9)	2452 (7.4)	843 (10.0)	8051 (7.9)	1428 (7.3)	628 (9.2)	5761 (9.4)	3655 (6.4)	691 (7.5)
BMI, mean (SD)	26.6 (4.4)	26.8 (4.4)	27.6 (4.9)	26.4 (4.2)	27.8 (4.7)	29.3 (5.5)	27.0 (4.6)	26.5 (4.2)	26.5 (4.2)
Physical activity, MET-min/wk, mean (SD)	2495.9 (2447.1)	2464.4 (2430.2)	2687.8 (2746.7)	2511.9 (2473.0)	2439.4 (2394.6)	2502.0 (2522.9)	2555.2 (2555.8)	2435.3 (2357.6)	2541.2 (2483.1)
Comorbidity									
Hypertension	20 014 (23.2)	7738 (23.4)	2057 (24.5)	22 929 (22.6)	4911 (25.3)	1969 (28.7)	14 642 (23.9)	13 059 (22.8)	2108 (22.7)
Diabetes	3142 (3.6)	926 (2.8)	276 (3.3)	2836 (2.8)	964 (5.0)	544 (7.9)	2498 (4.1)	1639 (2.9)	207 (2.2)
Cardiovascular disease	3221 (3.7)	1348 (4.1)	318 (3.8)	3815 (3.8)	774 (4.0)	298 (4.4)	2445 (4.0)	2090 (3.6)	352 (3.8)
Use of medication									
Antihypertensive drug	12 847 (14.9)	4899 (14.8)	1294 (15.4)	14 538 (14.3)	3170 (16.3)	1332 (19.4)	9374 (15.3)	8340 (14.5)	1326 (14.3)
RAAS inhibitor	7802 (9.0)	3014 (9.1)	806 (9.6)	8725 (8.6)	2023 (10.4)	874 (12.8)	5649 (9.2)	5147 (9.0)	826 (8.9)
Statin	10 466 (12.1)	3832 (11.6)	965 (11.5)	11 692 (11.5)	2579 (13.3)	992 (14.5)	7558 (12.4)	6653 (11.6)	1052 (11.3)
Laboratory findings									
eGFR, ml/min/1.73 m^2^	95.9 (11.5)	95.9 (11.7)	97.2 (12.0)	95.8 (11.5)	96.6 (11.7)	97.9 (11.9)	96.0 (11.7)	95.9 (11.5)	96.9 (11.5)
Triglyceride, mg/dL	144.9 (84.0)	151.6 (88.6)	160.6 (94.6)	146.3 (84.7)	151.5 (89.1)	157.3 (95.7)	146.8 (86.9)	147.8 (84.7)	152.7 (89.1)
LDL-C, mg/dL	138.1 (32.5)	138.5 (32.5)	136.9 (32.1)	138.6 (32.4)	137.2 (32.7)	134.5 (32.8)	137.7 (32.6)	138.6 (32.3)	138.2 (32.2)
HDL-C, mg/dL	57.6 (14.9)	55.9 (14.3)	53.1 (13.7)	57.3 (14.8)	55.6 (14.3)	53.8 (14.1)	56.8 (14.8)	57.2 (14.6)	55.8 (14.6)
Fasting glucose, mg/dL	91.0 (18.3)	90.6 (17.9)	91.0 (20.5)	90.6 (17.0)	91.6 (21.1)	93.6 (26.8)	91.1 (19.3)	90.8 (17.3)	90.7 (18.2)
hsCRP, mg/dL	0.21 (0.38)	0.22 (0.38)	0.24 (0.39)	0.21 (0.37)	0.24 (0.39)	0.27 (4.2)	0.23 (0.38)	0.21 (0.37)	0.21 (0.38)
Urine albumin to creatinine ratio, mg/g	11.2 (6.4)	10.6 (6.2)	10.1 (6.1)	11.0 (6.4)	10.8 (6.3)	10.8 (6.4)	11.0 (6.4)	11.0 (6.4)	10.7 (6.3)
Total energy intake, kJ/d	8416.2 (2238.3)	8817.6 (2082.5)	9557.6 (2348.4)	8626.8 (2243.3)	8469.8 (2115.4)	8477.4 (2297.1)	8320.1 (2310.5)	8760.7 (2094.2)	9381.7 (2189.9)
Total sugar intake, g/d	117.1 (45.0)	132.5 (43.2)	168.4 (53.1)	125.0 (47.1)	122.2 (44.9)	122.7 (52.0)	113.5 (46.8)	130.1 (42.6)	162.2 (50.0)
Sugar-sweetened beverages, servings/d	0.0 (0)	0.6 (0.3)	2.2 (0.9)	0.3 (0.6)	0.3 (0.6)	0.4 (0.8)	0.3 (0.7)	0.3 (0.6)	0.3 (0.7)
Artificially sweetened beverages, servings/d	0.2 (0.6)	0.2 (0.5)	0.3 (0.7)	0.0 (0)	0.6 (0.3)	2.3 (1.0)	0.3 (0.7)	0.2 (0.5)	0.2 (0.5)
Natural juice, servings/d	0.4 (0.6)	0.4 (0.5)	0.4 (0.6)	0.4 (0.6)	0.4 (0.5)	0.3 (0.6)	0.0 (0)	0.7 (0.3)	1.8 (0.7)
Water, servings/d	2.4 (1.5)	2.1 (1.4)	2.1 (1.5)	2.3 (1.5)	2.2 (1.4)	2.2 (1.5)	2.3 (1.6)	2.3 (1.4)	2.4 (1.5)
Healthy diet score^d^	4.1 (1.4)	3.9 (1.4)	3.7 (1.5)	4.1 (1.4)	4.0 (1.4)	3.8 (1.4)	3.9 (1.4)	4.1 (1.4)	4.2 (1.4)
Vegetable intake, servings/d	5.0 (3.3)	4.7 (3.0)	4.6 (3.4)	4.9 (3.2)	4.9 (3.2)	5.0 (3.4)	4.9 (3.4)	4.8 (3.0)	5.1 (3.6)
Fruit intake, servings/d	3.2 (2.5)	3.0 (2.4)	2.9 (2.6)	3.2 (2.5)	3.1 (2.4)	3.0 (2.4)	3.0 (2.4)	3.2 (2.4)	3.6 (3.0)
Fish intake, servings/wk	2.3 (1.6)	2.2 (1.5)	2.1 (1.6)	2.3 (1.6)	2.2 (1.5)	2.1 (1.6)	2.2 (1.6)	2.3 (1.5)	2.4 (1.7)
Processed meats intake, servings/wk	1.4 (1.4)	1.5 (1.4)	1.7 (1.5)	1.4 (1.4)	1.5 (1.4)	1.6 (1.5)	1.5 (1.4)	1.5 (1.4)	1.4 (1.4)
Red meat intake, servings/wk	2.0 (1.4)	2.1 (1.4)	2.1 (1.5)	2.0 (1.4)	2.1 (1.4)	2.1 (1.4)	2.1 (1.4)	2.1 (1.3)	2.0 (1.4)
Whole grain intake, servings/d	8.5 (9.0)	8.1 (8.9)	7.4 (9.1)	8.5 (9.1)	7.9 (8.6)	7.5 (8.7)	7.8 (9.0)	8.8 (8.9)	9.1 (9.3)
Refined grain intake, servings/d	6.4 (7.0)	6.9 (7.5)	7.8 (8.3)	6.6 (7.2)	6.5 (7.3)	6.6 (7.7)	6.8 (7.7)	6.5 (6.9)	6.3 (6.9)

Abbreviations: BMI, body mass index (calculated as weight in kilograms divided by height in meters squared); eGFR, estimated glomerular filtration rate; HDL-C, high-density lipoprotein cholesterol; hsCRP, high-sensitive C-reactive protein; LDL-C, low-density lipoprotein cholesterol; MET, metabolic equivalent; RAAS, renin-angiotensin-aldosterone system.

SI conversion factors: To convert glucose to millimoles per liter, multiply values by 0.0555; high-density lipoprotein cholesterol and low-density lipoprotein cholesterol to millimoles per liter, by 0.0259; hsCRP to milligrams per liter, by 10; serum triglyceride levels to millimoles per liter, by 0.0113.

^a^ The values for categorical variables are given as numbers (percentage) and values for continuous variables are given as mean (standard deviation).

^b^ Multiethnic or other group includes Black and White Caribbean, Black and White African, Asian and White, other mixed background, mixed, or other ethnic group.

^c^ €1.00 is approximately US $1.10.

^d^ Healthy diet score was calculated based on 7 dietary factors according to recommendations for dietary priorities on cardiometabolic health and ranged from 0 to 7. Each favorable dietary factor contributed 1 point to the overall score: total vegetables, 4 or more servings per day; total fruit, 4 or more servings per day; total fish, 2 or more servings per week; processed meat, 1 or fewer servings per week; red meat, 1.5 or fewer servings per week; whole grains, 3 or more servings per day; refined grains, 1.5 or fewer servings per day.

### Association of the Consumption of 3 Types of Beverages With Incident CKD

**Figure 2.**
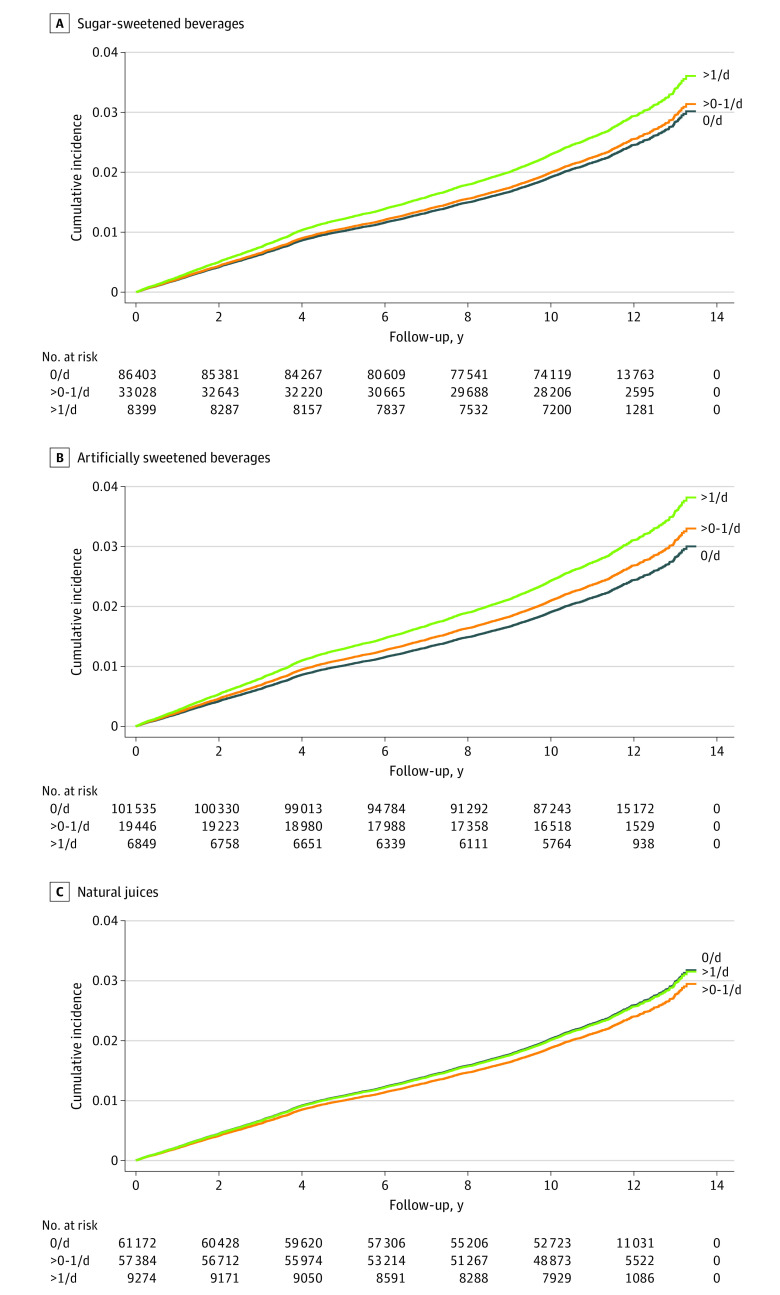
Adjusted Survival Curves for Incident Chronic Kidney Disease According to Category of Beverage and Level of Intake Curves are adjusted for age, sex, ethnic background, Townsend Deprivation Index, alcohol consumption status, smoking status, body mass index, physical activity, comorbidity (hypertension, diabetes, and cardiovascular disease), use of medication (renin-angiotensin-aldosterone system inhibitor and statins), dietary intake (total energy, total sugar, and healthy diet score), and laboratory measurements (estimated glomerular filtration rate, urine albumin to creatinine ratio, low-density lipoprotein cholesterol, and high-sensitive C-reactive protein).

In total, 4459 cases of incident CKD occurred during the median (IQR) follow-up of 10.5 (10.4-11.2) years. In multivariable Cox proportional hazards models, the AHR for participants with more than 1 serving per day of sugar-sweetened beverages was 1.19 (95% CI, 1.05-1.34), compared with participants who did not consume sugar-sweetened beverages. There was a significant dose-response association between the consumption of sugar-sweetened beverages and CKD development (HR, 1.04 95% CI, 0.97-1.12 for >0-1 serving per day; *P* = .006 for trend) (Table 2). The adjusted survival curve also showed that the risk of incident CKD was higher in participants with 1 serving per day of sugar-sweetened beverages (Figure 2A). When adjusting for confounding factors, the AHR for participants consuming artificially sweetened beverages of more than 0 to 1 serving per day was 1.10 (95% CI, 1.01-1.20) and for more than 1 serving per day was 1.26 (95% CI, 1.12-1.43) compared with the participants who did not consume artificially sweetened beverages (*P* < .001 for trend) (Table 2). The adjusted survival showed that participants in the highest artificially sweetened beverages consumption category (>1 serving per day) showed a higher incidence of CKD than participants in the lower consumption of artificially sweetened beverages categories (>1 vs 0 servings per day, AHR, 1.26 [95% CI, 1.12-1.43]; >1 vs >0-1 servings per day, AHR, 1.15 [95% CI, 1.01-1.32]). (Figure 2B). However, there was no significant association between natural juice intake and incident CKD (eg, for >1 serving per day, HR, 0.99 [95% CI, 0.87-1.11]; *P* = .10 for trend) (Table 2 and Figure 2C).

**Table 2.** Risk of Incident Chronic Kidney Disease by Category of Beverage Intake

Incident chronic kidney disease^a^^,^^b^	0 serving/d, HR (95% CI)	*P* value	>0-1 serving/d, HR (95% CI)	*P* value	>1 serving/d, HR (95% CI)	*P* value	*P* value for trend
**Sugar sweetened beverages**							
Person-years	905 494	NA	337 862	NA	87 613	NA	NA
Cases/events	2991/86 403	NA	1144/33 028	NA	324/8399	NA	NA
Model 1	1 [Reference]	NA	1.09 (1.02-1.17)	.01	1.42 (1.26-1.59)	<.001	<.001
Model 2	1 [Reference]	NA	1.08 (1.01-1.16)	.03	1.28 (1.14-1.43)	<.001	<.001
Model 3	1 [Reference]	NA	1.04 (0.97-1.12)	.26	1.19 (1.05-1.34)	.01	.006
**Artificially sweetened beverages**							
Person-years	1 061 369	NA	198 621	NA	70 979	NA	
Cases/events	3470/101 535	NA	695/19 446	NA	294/6849	NA	
Model 1	1 [Reference]	NA	1.27 (1.17-1.37)	<.001	1.70 (1.51-1.92)	<.001	<.001
Model 2	1 [Reference]	NA	1.10 (1.02-1.20)	.02	1.24 (1.10-1.40)	.001	<.001
Model 3	1 [Reference]	NA	1.10 (1.01-1.20)	.02	1.26 (1.12-1.43)	<.001	<.001
**Natural juices**							
Person-years	645 516	NA	589 535	NA	95 917	NA	NA
Cases/events	2277/61 172		1871/57 384	NA	311/9274	NA	NA
Model 1	1 [Reference]	NA	0.85 (0.80-0.91)	.001	0.92 (0.81-1.03)	.15	<.001
Model 2	1 [Reference]	NA	0.92 (0.87–0.98)	.01	0.98 (0.87-1.10)	.70	.07
Model 3	1 [Reference]	NA	0.93 (0.87-0.99)	.03	0.99 (0.87-1.11)	.90	.10

Abbreviations: HR, hazard ratio; NA, not applicable.

^a^ Model 1 adjusted for age, sex. Model 2 adjusted as for model 1 plus ethnic background, Townsend Deprivation Index, alcohol consumption status, smoking status, body mass index, physical activity, comorbidity (hypertension, diabetes, and cardiovascular disease), and the use of medication (renin-angiotensin-aldosterone system inhibitor and statins). Model 3 adjusted as for model 2 plus dietary intake (total energy, total sugar, and healthy diet score) and laboratory measurements (estimated glomerular filtration rate, urine albumin to creatinine ratio, low-density lipoprotein cholesterol, and high-sensitive C-reactive protein).

^b^ Healthy diet score was calculated based on 7 dietary factors according to recommendations for dietary priorities on cardiometabolic health and ranged from 0 to 7. Each favorable dietary factor contributed 1 point to the overall score: total vegetables 4 or more servings per day; total fruit 4 or more servings per day; total fish 2 or more servings per week; processed meat 1 or fewer servings per week; red meat 1.5 or fewer servings per week; whole grains 3 or more servings per day; refined grains 1.5 or fewer servings per day.

The results of analyses using a subcohort with a composite CKD outcome were consistent with the main analyses. The baseline characteristics of the subcohort are shown in eTable 3 in Supplement 1. There was a similar association between beverage intake and the risk of composite CKD outcome (eTable 4 in Supplement 1).

### Association Between Replacing 1 Serving per Day of a Specific Type of Beverage and Incident CKD

In substitution analyses, there was no significant risk difference in incident CKD when replacing sugar-sweetened beverages with artificially sweetened beverages (HR, 1.03 [95% CI, 0.96-1.10]). Replacing 1 serving per day of sugar-sweetened beverages with natural juices was associated with 6.7% lower risk of incident CKD (HR, 0.93 [95% CI, 0.87-0.97]), and replacing 1 serving per day of sugar-sweetened beverages with water was also associated with 6.7% lower risk (HR, 0.93 [95% CI, 0.88-0.99]). Similarly, replacing 1 serving per day of artificially sweetened beverages with natural juices was associated with 9.9% lower risk of CKD (HR, 0.90 [95% CI, 0.84-0.96]) and with water was associated with 8.6% lower risk of CKD (HR, 0.91 [95% CI, 0.86-0.96]) (Table 3).

**Table 3.** Substitution Analysis Examining the Association Between Risk of Incident Chronic Kidney Disease and Category of Beverage Intake

Substitution analysis^a^	Sugar-sweetened beverages, HR (95% CI)	*P* value	Artificially sweetened beverages, HR (95% CI)	*P* value	Natural juices, HR (95% CI)	*P* value
With sugar-sweetened beverages	1 [Reference]	NA	0.97 (0.91-1.04)	.36	1.08 (1.01-1.15)	.04
With artificially sweetened beverages	1.03 (0.96-1.10)	.36	1 [Reference]	NA	1.11 (1.04-1.20)	.003
With natural juices	0.93 (0.87-0.97)	.04	0.90 (0.84-0.96)	.003	1 [Reference]	NA
With water	0.93 (0.88-0.99)	.03	0.91 (0.86-0.96)	.001	1.04 (0.97-1.11)	.23

Abbreviations: HR, hazard ratio; NA, not applicable.

^a^ Healthy diet scores as in the footnote to Table 2. Model adjusted for age, sex, ethnic background, Townsend Deprivation Index, alcohol consumption status, smoking status, body mass index, physical activity, comorbidity (hypertension, diabetes, and cardiovascular disease), the use of medication (renin-angiotensin-aldosterone system inhibitor and statins), dietary intake (total energy, total sugar, and healthy diet score), and laboratory measurements (estimated glomerular filtration rate, urine albumin to creatinine ratio, low-density lipoprotein cholesterol, and high-sensitive C-reactive protein).

### Mediation Analysis

Sugar intake (β coefficient, 0.03 [95% CI, 0.00-0.06]; percentage, 18.7%) and BMI (β coefficient, 0.03 [95% CI, 0.03-0.04]; percentage, 20.0%) partly mediated the association between sugar-sweetened beverages and the risk of CKD. On the other hand, BMI (β coefficient, 0.09 [95% CI, 0.08-0.11]; percentage, 27.3%) showed an indirect effect on the association between artificially sweetened beverages and CKD development (eTable 5 in Supplement 1).

### Sensitivity and Subgroup Analyses

The association between beverage consumption and CKD risk in sensitivity analyses remained robust. A consistent association was observed when (1) using data from the first dietary assessment as the primary exposure (eTable 6 in Supplement 1); (2) excluding participants who developed CKD events in the first 3 years of the follow-up period (eTable 7 in Supplement 1); (3) including participants who completed at least 2 dietary assessments (eTable 8 in Supplement 1); and (4) adjusting for household income level rather than Townsend Deprivation Index (eTable 9 in Supplement 1). Furthermore, after the Townsend Deprivation Index score, alcohol consumption status, smoking status, BMI, physical activity, low-density lipoprotein cholesterol level, and hsCRP were imputed, the results remained consistent across different imputation methods (eTable 10 in Supplement 1).

Finally, we assessed the interactions between beverage intake and prespecified subgroups on the risk of the CKD outcome. There were no significant interactions between most subgroup factors and beverage intake for incident CKD. However, there was an interaction between sex and natural juice intake on the risk of incident CKD (eFigure in Supplement 1).

## Discussion

Using data from the large UK Biobank cohort, this cohort study examined the association between the consumption of 3 different types of beverage and the risk of CKD. Our findings indicated that consuming more than 1 serving per day of sugar-sweetened beverages or any amount of artificially sweetened beverages was associated with increased risk of incident CKD. Although natural juice consumption itself was not associated with the risk of incident CKD, substituting 1 serving per day of sugar-sweetened beverages or artificially sweetened beverages with natural juices or water was associated with reduced risk of incident CKD. These findings may offer insight into the association between beverage intake and the prevention of CKD.

Previous studies^12,13,14,15,16,22^ have found that the associations between the consumption of different beverage types and CKD risk are inconsistent. The Atherosclerosis Risk in Communities Study did not find any association between high consumption of sugar-sweetened beverages and CKD development,^13^ whereas a significant association was found between artificially sweetened beverage intake and the risk of end-stage kidney disease in the same study.^22^ Similarly, a study of 3318 women without kidney dysfunction did not show a significant association between consumption of sugar-sweetened beverages and kidney function decline but showed that the consumption of 2 or more servings per day of artificially sweetened beverages was associated with 2-fold increased odds for kidney function decline.^14^ In contrast, in the Tehran Lipid and Glucose Study of 1690 participants without CKD, consuming sugar-sweetened beverages was associated with higher odds of incident CKD.^12^ Furthermore, based on principal components analysis involving many types of beverages (sugar-sweetened beverages, artificially sweetened beverages, juices, tea, coffee, alcohol, and water), a pattern of consuming sugar-sweetened beverages was associated with increased odds of CKD in a community-based cohort of black Americans.^16^ These conflicting results may be due to the heterogeneity of cohort characteristics, study design, sample sizes, assessments of dietary intake, and the definition of CKD outcome. However, a recent meta-analysis reported a modest but statistically significant increase in CKD risk when consuming above 7 servings per week of sugar-sweetened beverages or artificially sweetened beverages in dose-response analysis.^15^ Our results align with this finding, showing an increased CKD risk among participants who consumed more than 1 serving per day of sugar-sweetened beverages or more than 0 to 1 and more than 1 serving per day of artificially sweetened beverages. In addition, replacing sugar-sweetened beverages or artificially sweetened beverages with natural juices was associated with a decreased CKD risk in the present study. These findings highlight the importance of considering the potential adverse effects of sugar-sweetened beverages and artificially sweetened beverages on kidney health. Given the conflicting findings and limitations of previous studies, our research contributes to the growing evidence supporting the restriction of both sugar-sweetened beverages and artificially sweetened beverages to prevent CKD.

Sugar-sweetened beverages, recognized as a major source of free sugars, are associated with a high glycemic load, leading to increased blood glucose and hyperinsulinemia.^23^ A high glycemic load can contribute to diabetes-related metabolic alterations, such as glucose intolerance and insulin resistance,^24,25^ and significant weight gain.^26^ The high sugar content in sugar-sweetened beverages, particularly fructose, can lead to glomerular hyperfiltration and accelerate the decline of kidney function.^27^ Animal models have also suggested that fructose can trigger inflammatory and oxidative stress responses, altering the intestinal microbiota.^28^ In addition, both sugar-sweetened beverages and artificially sweetened beverages are high in phosphorus and dietary acid, which are known risk factors for kidney disease.^29^ Consistent with these previous studies, the results of our mediation analysis showed an indirect association with sugar intake and BMI.

Artificially sweetened beverages have gained popularity as alternatives to sugar-sweetened beverages, especially among individuals concerned about metabolic syndrome and obesity-related complications.^30^ However, the present study found that substituting sugar-sweetened beverages with artificially sweetened beverages offered no additional benefit in CKD prevention. Rather, artificially sweetened beverage intake was associated with an increased risk of CKD development, and BMI had an indirect effect on the association between the intake of artificially sweetened beverages and CKD in our mediation analysis. Previous studies have suggested that long-term intake of artificial sweeteners may increase adipose tissue accumulation and lead to weight gain, independent of caloric intake.^31^ One plausible mechanism is that artificially sweetened beverage consumption could alter the composition and function of the gut microbiota.^32^ In addition, the decreased satiety and increased preference for sweet flavors associated with artificial sweetener intake could affect overall dietary patterns.^33^ In rat models, the ingestion of both low and high doses of saccharin resulted in increased levels of serum urea and creatinine and increased sodium excretion.^34,35^ Furthermore, long-term aspartame intake is associated with increased free radical production in kidney tissues, contributing to kidney injury.^36^

Natural juices did not show a significant association with CKD risk in this study. The reason natural juice consumption did not show a favorable association may be due to the large amount of sugar in natural juices, similar to that in sugar-sweetened beverages. Although natural juice consumption did not increase the risk of CKD development, excessive consumption of natural juices should be avoided in high-risk individuals because it can increase the risk of various metabolic diseases that are risk factors for CKD. Nevertheless, replacing sugar-sweetened beverages or artificially sweetened beverages with natural juices significantly lowered CKD risk. This is likely because natural juices are rich in beneficial nutrients, such as vitamin C and potassium, potentially lowering blood pressure and alleviating the inflammatory process.^24,37^

### Strengths and Limitations

The strengths of this study include our use of data from UK Biobank, a large prospective cohort study that includes various confounding factors. Moreover, different sensitivity and subgroup analyses showed robust findings. This study also has limitations. First, owing to the observational nature, a causal relationship between beverage intake and CKD development could not be determined. Second, because we used data from 24-hour recall dietary assessments, there was the potential for misreporting dietary intake and misclassifying beverage types, and this dietary assessment could not establish a relationship between the absolute amount of beverage intake and CKD. Moreover, a single 24-hour recall dietary assessment could not capture the changes in dietary patterns and habitual intake. However, the results were similar when including participants who completed the dietary assessment at least twice. Third, incident CKD was defined based on claims codes, potentially leading to misclassification. Furthermore, there can be variations in the accuracy of diagnosis based on different data sources. To address these concerns, we conducted a sensitivity analysis using follow-up creatinine values from general practice and UK biobank data and showed similar results, suggesting robustness in our findings. However, considering the limited availability of follow-up creatinine data in this study, there is a possibility of misclassification of the CKD outcome, which could potentially impact the accuracy of our findings. Fourth, while this study focused on examining the association between beverage intake and CKD development, we did not estimate the potential associations with specific types or contents of sugary and artificial sweeteners. Additionally, while this study conducted mediation analyses for some aspects, we observed only a partial indirect effect associated with sugar intake or BMI. Therefore, further research using metabolites or biomarkers is needed to fully explain the complex association between beverage consumption and risk of CKD. Finally, UK Biobank includes middle-aged, predominantly white participants, which may limit the generalizability of our findings.

## Conclusions

In this cohort study, higher consumption of sugar-sweetened beverages or artificially sweetened beverages was associated with higher risk of incident CKD, while the consumption of natural juices did not increase the risk of incident CKD. These results suggest that healthy beverage consumption habits may be important for preventing CKD.

## Notes

Supplement 1. **eMethods 1.** Data collection and measurements **eMethods 2.** Outcome measures **eMethods 3.** Statistical analyses- Cox proportional hazards model **eMethods 4.** Statistical analyses- substitution and mediation analyses **eMethods 5.** Statistical analyses- imputation method **eMethods 6.** Statistical analyses- subgroup analyses **eTable 1.** Number and percentages of participants who completed the 24-hour dietary assessment in UK Biobank cohort study **eTable 2.** Definitions and codes used for defining chronic kidney disease **eTable 3.** Baseline characteristics by availability of follow up creatinine results in linked general practice or UK Biobank data **eTable 4.** Risk of composite chronic kidney disease development by category of beverage intake **eTable 5.** Risk of incident chronic kidney disease by category beverage intake with four-way decomposition by sugar intake and BMI **eTable 6.** Risk of incident chronic kidney disease by category of beverage intake from first completed dietary questionnaire **eTable 7.** Risk of incident chronic kidney disease by category of beverage intake, excluding participants developing events during first 3 years of follow-up **eTable 8.** Risk of incident chronic kidney disease by category of beverage intake among the participants who conducted two or more dietary assessments **eTable 9.** Risk of incident chronic kidney disease by category of beverage intake by adjustment of household income **eTable 10.** Risk of incident chronic kidney disease by category of beverage intake after using median imputation, missing indicator methods and multiple imputation by chain equation **eFigure.** Multivariable-adjusted hazard ratios for incident chronic kidney disease stratified by subgroup

Supplement 2.
Data Sharing Statement
