# Supplementary material for: Sweetened Beverage Intake and Incident Chronic Kidney Disease in the UK Biobank Study
Source: JAMA Netw Open. 2024 Feb 28;7(2):e2356885. doi: 10.1001/jamanetworkopen.2023.56885 (PMC10902724; doi:10.1001/jamanetworkopen.2023.56885)
Supplement: Supplement 1. — eMethods 1. Data collection and measurements eMethods 2. Outcome measures eMethods 3. Statistical analyses- Cox proportional hazards model eMethods 4. Statistical analyses- substitution and mediation analyses eMethods 5. Statistical analyses- imputation method eMethods 6. Statistical analyses- subgroup analyses eTable 1. Number and percentages of participants who completed the 24-hour dietary assessment in UK Biobank cohort study eTable 2. Definitions and codes used for defining chronic kidney disease eTable 3. Baseline characteristics by availability of follow up creatinine results in linked general practice or UK Biobank data eTable 4. Risk of composite chronic kidney disease development by category of beverage intake eTable 5. Risk of incident chronic kidney disease by category beverage intake with four-way decomposition by sugar intake and BMI eTable 6. Risk of incident chronic kidney disease by category of beverage intake from first completed dietary questionnaire eTable 7. Risk of incident chronic kidney disease by category of beverage intake, excluding participants developing events during first 3 years of follow-up eTable 8. Risk of incident chronic kidney disease by category of beverage intake among the participants who conducted two or more dietary assessments eTable 9. Risk of incident chronic kidney disease by category of beverage intake by adjustment of household income eTable 10. Risk of incident chronic kidney disease by category of beverage intake after using median imputation, missing indicator methods and multiple imputation by chain equation eFigure. Multivariable-adjusted hazard ratios for incident chronic kidney disease stratified by subgroup [file jamanetwopen-e2356885-s001.pdf]

Supplementary Online Content

Heo GY, Koh HB, Park JT, et al. Sweetened beverage intake and chronic kidney disease in the UK Biobank study. *JAMA Netw Open*. 2024;7(2):e2356885.
doi:10.1001/jamanetworkopen.2023.56885

eMethods 1. Data collection and measurements

eMethods 2. Outcome measures

eMethods 3. Statistical analyses- Cox proportional hazards model

eMethods 4. Statistical analyses- substitution and mediation analyses

eMethods 5. Statistical analyses- imputation method

eMethods 6. Statistical analyses- subgroup analyses

eReferences.

eTable 1. Number and percentages of participants who completed the 24-hour dietary assessment in UK Biobank cohort study

eTable 2. Definitions and codes used for defining chronic kidney disease

eTable 3. Baseline characteristics by availability of follow up creatinine results in linked general practice or UK Biobank data

eTable 4. Risk of composite chronic kidney disease development by category of beverage intake

eTable 5. Risk of incident chronic kidney disease by category of beverage intake with four-way decomposition by sugar intake and BMI

eTable 6. Risk of incident chronic kidney disease by category of beverage intake from first completed dietary questionnaire

eTable 7. Risk of incident chronic kidney disease by category of beverage intake, excluding participants developing events during first 3 years of follow-up

eTable 8. Risk of incident chronic kidney disease by category of beverage intake among the participants who conducted two or more dietary assessments

eTable 9. Risk of incident chronic kidney disease by category of beverage intake by adjustment of household income

eTable 10. Risk of incident chronic kidney disease by category of beverage intake after using median imputation, missing indicator methods and multiple imputation by chain equation

© 2024 Heo GY et al. *JAMA Network Open*.

eFigure. Multivariable-adjusted hazard ratios for incident chronic kidney disease stratified by subgroup

This supplementary material has been provided by the authors to give readers additional information about their work.

© 2024 Heo GY et al. *JAMA Network Open*.

eMethods 1. Data collection and measurements

The detailed study protocols have been described in a previous study.[1] Sociodemographic data, lifestyle information, and medical history were collected at recruitment. Ethnicity was categorized as 'Asian', 'Black', 'Multiethnic or other' and 'White', based on self-reported data. The Townsend deprivation index was used to measure socioeconomic deprivation, based on the participants' residential postcode, incorporating the information on employment status, ownership of a car and home, and household crowding Household income (£/year) was self-reported as following: (<18 000; 18,000 to 30,999; 31,000 to 51,999; 52,000 to 100,000; >100,000). Smoking and alcohol consumption status was classified into three categories as follows: "never," "previous," and "current." Body mass index (BMI) was calculated by dividing weight by height squared (kg/m^2). Physical activity was measured in metabolic equivalent of task-minutes per week to sum up all types of activities, such as walking, moderate and vigorous activity.[2] Comorbidities were defined as self-reported physician-diagnosed cases or International Classification of Diseases-10th Revision (ICD-10) codes as follows: hypertension (I10-13 and I15), diabetes (E10-14), cardiovascular disease (CVD) (I20-25 and I60-64), and malignancy (C00-97). The use of antihypertensive and antidiabetic drugs defined as hypertension and diabetes, respectively. The use of medication, including renin-angiotensin-aldosterone system (RAAS) blockers and statins was identified based on verbal interview data.

Blood and urine samples were collected at recruitment. Serum creatinine was measured by the isotope dilution mass spectrometry-traceable method, and the CKD Epidemiology Collaboration creatinine equation was used to calculate eGFR.[3] Among one-third of the participants, follow-up serum creatinine values were obtained from UK Biobank follow-up testing and linked GP records. Urine creatinine was measured by the enzymatic method, and urine albumin was measured by the immune-turbidimetric method. Other laboratory data measurement methods were as follows: serum glucose, total cholesterol, triglyceride (enzymatic), high-density lipoprotein cholesterol (enzyme immune-inhibition), low-density lipoprotein cholesterol (LDL-C) (enzymatic selective protection), and high-sensitivity C-reactive protein (hs-CRP) levels (Immuno-turbidimetric). Detailed methods for other laboratory measurements were described in the previous study protocol.[4]

Dietary information was collected up to five times between April 2009 and June 2012. Dietary information was collected using the Oxford WebQ, a web-based, self-administered 24-hour recall questionnaire. Oxford WebQ has been developed for large-scale population studies and validated against an interviewer-administered questionnaire.[5] This questionnaire collected information on 206 food and 32 beverage types consumed during the previous 24 hours.[6] The intake of the total energy and each macronutrient and micronutrient was estimated by the UK Biobank using a method previously described.[6] The quantity of food was obtained using the standard portion sizes in the UK Nutrient Databank Food Composition Table.[7] To capture the overall dietary pattern, we utilized the healthy diet score, based on seven dietary factors and cut-offs according to recommendations for dietary priorities on cardiometabolic health.[8,9] The score ranged from 0 to 7, which each favorable dietary factor contributed one point to the overall score; total fruit ≥ 4 servings/day; total vegetables ≥4 servings/day; total fish ≥2 servings/week; processed meat ≤1 serving/week; red meat ≤1.5 servings/week; whole grains ≥3 servings/day; refined grains ≤1.5 servings/day.

eMethods 2. Outcome measures

The primary outcome of the study was incident CKD, defined using International Classification of Diseases, 10th Edition (ICD-10) codes in hospital inpatient data and death register records, clinical codes in primary care data mapped to ICD-10 using Coding system lookups and mappings[10]or Office of Population Censuses and Surveys Classification of Interventions and Procedures (OPCS-4) codes from hospital inpatient data (**eTable 2**).

In the subcohort dataset, composite CKD outcome was used based on ICD-10 codes, OPSC-4 codes, clinical codes, or measurements of eGFR <60 mL/min/1.73 m^2, whichever came first. Deaths from any cause were ascertained by the National Health Service Information Centre for England and Wales and the National Health Service Central Register for Scotland. The outcomes were assessed from the date each participant completed the last dietary questionnaire. The last follow-up was until October 31, 2022, for participants in England, until July 31, 2021, for participants in Scotland, and until February 28, 2018, for participants in Wales. CKD outcomes were assessed from when the participants completed the 24-hour dietary questionnaire to the date of CKD development, death, or the last follow-up, whichever came first.

© 2024 Heo GY et al. *JAMA Network Open*.

eMethods 3. Statistical analyses- Cox proportional hazards model

The primary analysis used the Cox proportional hazard model. The proportionality assumption was examined using the Schoenfeld residuals method.[11] Linear trends were calculated using each category as a continuous variable in the Cox model. Model 1 was minimally adjusted for age and sex. Model 2 was further adjusted for ethnic background, Townsend deprivation index, BMI, alcohol consumption status, smoking status, physical activity, comorbidities (hypertension, diabetes, and cardiovascular disease), and the use of medications (renin-angiotensin-aldosterone system blockers and statins). Model 3 further included dietary intake (total energy, sugar intake, and healthy diet score) and laboratory measurements (eGFR, LDL-C, UACR, and hs-CRP). The results were presented as adjusted hazard ratios (aHRs) and 95% confidence intervals (CIs).

eMethods 4. Statistical analyses- substitution and mediation analyses

Substitution analyses

This conducted a substitution analysis to evaluate the effect of substituting one beverage for another.[12] This analysis included two types of beverages in the same model. It estimated the difference in the hazard for a 1 serving/day increased intake of one type of beverage and a concomitantly decreased intake of another type of beverage.[12]

Mediation analyses

Causal mediation analysis with survival outcome were conducted using the STATA command, med4way, which decomposes the total effect into four components: (1) controlled direct effect, (2) reference interaction, (4) mediated interaction, and (4) pure indirect effect. Cox regression was conducted for a model of the exposure and outcome and linear regression was selected for a model of the exposure on the mediator. The association between beverage intake and CKD at mean levels of the mediator were tested. The proportion was defined as the ratio of each effect (direct effect, reference interaction, mediated interaction, indirect effect) to the total effect. Model was adjusted age, sex, ethnic background, Townsend deprivation index, BMI, alcohol consumption status, smoking status, physical activity, comorbidities (hypertension, diabetes, and cardiovascular disease), the use of medications (renin-angiotensin-aldosterone system blockers and statins), dietary intake (total energy, sugar intake, and healthy diet score) and laboratory measurements (eGFR, LDL-C, UACR and hs-CRP).

eMethods 5. Statistical analyses- imputation method

Missing values were identified in variables; Townsend deprivation index, alcohol consumption status, smoking status, body mass index, physical activity, low-density lipoprotein cholesterol, and high-sensitive C-reactive protein. Physical activity had the highest missing rate at 14.84%. However, the missing rates for other covariates were lower than 1%. As a single imputation method, we replaced missing values with the observed median of value. We also employed the missing indicator method, generating an additional binary variable (indicator variable) indicating a missing on the original variable and "0" indicating an observed value. This indicator variable is included in the analysis along with the original variable.[13,14] Furthermore, we used multiple imputations by chained equation under a 'missing at random' assumption and created five imputed datasets.[15] Predictive mean matching was used for continuous variables, and polytomous regression was used for categorical variables.[15] The HR was estimated using Rubin's formula.[16]

eMethods 6. Statistical analyses- subgroup analyses

We additionally examined whether the relationship between the three types of beverages and the development of CKD modified among prespecified subgroups by sex (male or female), age (<60 or ≥60 years), BMI (<25 or ≥25 kg/m^2), previous history of diabetes (yes or no), and hs-CRP (<1 or ≥1 mg/L).

© 2024 Heo GY et al. *JAMA Network Open*.

eReferences

1. Fry A, Littlejohns TJ, Sudlow C, et al. Comparison of Sociodemographic and Health-Related Characteristics of UK Biobank Participants With Those of the General Population. *Am J Epidemiol.* 2017;186(9):1026-1034, https://doi.org/10.1093/aje/kwx246.

2. *Guidelines for Data Processing and Analysis of the International Physical Activity Questionnaire (IPAQ)- short and long forms*. Accessed September 9, 2022. https://biobank.ndph.ox.ac.uk/ukb/ukb/docs/ipaq_analysis.pdf

3. Inker LA, Eneanya ND, Coresh J, et al. New Creatinine- and Cystatin C-Based Equations to Estimate GFR without Race. *N Engl J Med.* 2021;385(19):1737-1749, https://doi.org/10.1056/NEJMoa2102953.

4. UK Biobank. *Category 17518—Blood biochemistry,*September 9 2022. http://biobank.ctsu.ox.ac.uk/crystal/label.cgi?id=17518

5. Liu B, Young H, Crowe FL, et al. Development and evaluation of the Oxford WebQ, a low-cost, web-based method for assessment of previous 24 h dietary intakes in large-scale prospective studies. *Public Health Nutr.* 2011;14(11):1998-2005, https://doi.org/10.1017/s1368980011000942.

6. Perez-Cornago A, Pollard Z, Young H, et al. Description of the updated nutrition calculation of the Oxford WebQ questionnaire and comparison with the previous version among 207,144 participants in UK Biobank. *Eur J Nutr.* 2021;60(7):4019-4030, https://doi.org/10.1007/s00394-021-02558-4.

7. Swan G, Dodhia S, Farron-Wilson M, Powell N, Bush M. Food composition data and public health. *Nutr Bull.* 2015;40(3):223-226, https://doi.org/https://doi.org/10.1111/nbu.12156.

8. Lourida I, Hannon E, Littlejohns TJ, et al. Association of lifestyle and genetic risk with incidence of dementia. *Jama.* 2019;322(5):430-437.

9. Liu W, Wang T, Zhu M, Jin G. Healthy Diet, Polygenic Risk Score, and Upper Gastrointestinal Cancer Risk: A Prospective Study from UK Biobank. *Nutrients.* 2023;15(6), https://doi.org/10.3390/nu15061344.

10. biobank U. *Resource 592-Clinical coding classification systems and maps*. Accessed December 20, 2023.

11. GRAMBSCH PM, THERNEAU TM. Proportional hazards tests and diagnostics based on weighted residuals. *Biometrika.* 1994;81(3):515-526, https://doi.org/10.1093/biomet/81.3.515.

12. Ibsen DB, Laursen ASD, Würtz AML, et al. Food substitution models for nutritional epidemiology. *Am J Clin Nutr.* 2020;113(2):294-303, https://doi.org/10.1093/ajcn/nqaa315.

13. Greenland S. Theoretical Epidemiology: Principles of Occurrence Research in Medicine. *JAMA.* 1986;256(7):929-930, https://doi.org/10.1001/jama.1986.03380070135039.

14. Donders ART, van der Heijden GJMG, Stijnen T, Moons KGM. Review: A gentle introduction to imputation of missing values. *Journal of Clinical Epidemiology.* 2006;59(10):1087-1091, https://doi.org/https://doi.org/10.1016/j.jclinepi.2006.01.014.

15. Van Buuren S, Groothuis-Oudshoorn K. mice: Multivariate imputation by chained equations in R. *J Stat Softw.* 2011;45:1-67.

16. Lazar NA. Statistical analysis with missing data. Taylor & Francis; 2003.

© 2024 Heo GY et al. *JAMA Network Open*.

eTable 1. Number and percentages of participants who completed the 24-hour dietary assessment in UK Biobank cohort study

Number of response times	Number of participants, n (%)
1	50 349 (39.7)
2	30 033 (23.5)
3	26 159 (20.5)
4	18 021 (14.1)
5	3 268 (2.6)
**Median** 2.0 (**Interquartile range**, 1.0-3.0)	127 830

© 2024 Heo GY et al. *JAMA Network Open*.

eTable 2. Definitions and codes used for defining chronic kidney disease[a]

Hospital inpatient data, death register records and primary care data[a]	
**ICD-10 code**	**Description**
E102	Type 1 diabetes mellitus with kidney complications
E112	Type 2 diabetes mellitus with kidney complications
I12.x	Hypertensive chronic kidney disease
I13.x	Hypertensive heart and chronic kidney disease
N18.x	Chronic kidney disease.
T861	Complications of kidney transplant
Z940	Kidney transplant status
**OPCS-4 codes**	
L74.1-74.6	Insertion of arteriovenous prosthesis, creation of arteriovenous fistula, attention to arteriovenous shunt, banding of arteriovenous fistula, thrombectomy of arteriovenous fistula, creation of graft fistula for dialysis
L74.8-74.9	Other specified arteriovenous shunt, unspecified arteriovenous shunt
M01.2-01.9	Allotransplantation of kidney from live donor, allotransplantation of kidney from cadaver, allotransplantation of kidney from cadaver heart beating, allotransplantation of kidney from cadaver heart non-beating, other specified transplantation of kidney, unspecified transplantation of kidney
M02.3	Bilateral nephrectomy
M08.4	Exploration of transplanted kidney
M17.2	Pre-transplantation of kidney work-up – recipient
M17.4	Post-transplantation of kidney examination – recipient
M17.8-17.9	Other specified interventions associated with transplantation of kidney, unspecified interventions associated with transplantation of kidney
X40.2	Renal dialysis
X40.5-40.6	Peritoneal dialysis NEC, hemodialysis NEC, hemofiltration, automated peritoneal dialysis, continuous ambulatory peritoneal dialysis,
X41.1-41.2	Insertion of ambulatory peritoneal dialysis catheter, removal of ambulatory peritoneal dialysis catheter

Abbreviations: ICD-10, International Classification of Diseases-10th Revision; OPCS-4, Office of Population Censuses and Surveys Classification of Interventions and Procedures

[a] Incident CKD was defined using ICD-10 codes in hospital inpatient data and death register records, clinical codes in primary care data or OPCS-4 codes from hospital inpatient data. Clinical codes (read 2 or read 3) were mapped to ICD-10 using Coding system lookups and mappings, version 3.

© 2024 Heo GY et al. *JAMA Network Open*.

eTable 3. Baseline characteristics by availability of follow up creatinine results in linked general practice or UK biobank data

Characteristics[a]	Total N= 127 830	Availability of follow up creatinine	
		Non available N= 80 894	Available N=46 936
**Age, years**	55.2 (8.0)	55.0 (8.0)	55.6 (7.8)
**Female, n (%)**	66 180 (51.8)	42 115 (52.1)	24 065 (51.3)
**Ethnicity**			
Asian	2 055 (1.6)	1 333 (1.6)	722 (1.5)
Black	1 447 (1.1)	1 115 (1.4)	332 (0.7)
Multiethnic or other[b]	1 712 (1.3)	1 198 (1.5)	514 (1.1)
White	122 616 (95.9)	77 248 (95.5)	45 368 (96.7)
**Drinking status**			
Never	3 659 (2.9)	2 351 (2.9)	1 308 (2.8)
Previous	3 538 (2.8)	2 196 (2.7)	1 342 (2.9)
Current	120 633 (94.4)	76 347 (94.4)	44 286 (94.4)
**Smoking status**			
Never	73 161 (57.2)	46 280 (57.2)	26 881 (57.3)
Previous	44 562 (34.9)	28 114 (34.8)	16 448 (35.0)
Current	10 107 (7.9)	6 500 (8.0)	3 607 (7.7)
**BMI**	26.75 (4.42)	26.72 (4.43)	26.80 (4.40)
**Physical activity, MET-min/week**	2500.4 (2464.1)	2492.6 (2459.6)	2513.8 (2471.8)
**Total energy intake, kJ/day**	8594.9 (2228.1)	8584.7 (2232.2)	8612.5 (2221.1)
**Total sugar intake, g/day**	124.5 (47.1)	124.0 (47.0)	125.2 (47.2)
**Healthy diet score[c]**	4.0 (1.4)	4.0 (1.4)	4.0 (1.4)
**Comorbidity**			
Hypertension	29 809 (23.3)	19 440 (24.0)	10 369 (22.1)
Diabetes	29 809 (23.3)	19 440 (24.0)	10 369 (22.1)
Cardiovascular disease	4 887 (3.8)	3 021 (3.7)	1 866 (4.0)
**Medication use**			
RAAS blocker	11 622 (9.1)	8 162 (10.1)	3 460 (7.4)
Statin	15 263 (11.9)	9 614 (11.9)	5 649 (12.0)
**Laboratory findings**			
eGFR, ml/min/1.73 m^2	96.0 (11.6)	96.0 (11.7)	96.0 (11.4)
LDL-C, mg/dL	138.2 (32.5)	137.7 (32.3)	138.9 (32.6)
hs-CRP, mg/dL	0.22 (0.38)	0.22 (0.37)	0.22 (0.40)
Urine albumin to creatinine ratio, mg/g	11.0 (6.4)	11.0 (6.4)	11.0 (6.4)

Abbreviations: BMI, body mass index (calculated as weight in kilograms divided by height in meters squared); MET, metabolic equivalent; RAAS, renin-angiotensin-aldosterone system; eGFR, estimated glomerular filtration rate; LDL-C, low-density lipoprotein cholesterol; hs-CRP, high-sensitive C-reactive protein.

[a]The values for categorical variables are given as numbers (percentage) and values for continuous variables are given as mean (standard deviation).

[b] Multiethnic or other group includes White and Black and White Caribbean, White and Black and White African, White and Asian and White, other mixed background, mixed, or other. [c]Healthy diet scores as in the footnote to Table 2

© 2024 Heo GY et al. *JAMA Network Open*.

eTable 4. Risk of composite chronic kidney disease development by category of beverage intake

Composite chronic kidney disease[a]	0 serving/day HR (95% CI)	P value	>0-1 servings/day HR (95% CI)	P value	>1 servings/day HR (95% CI)	P value	P for trend
**Sugar sweetened beverages**							
Model 1	1 [reference]	NA	1.07 (0.98-1.17)	.15	1.24 (1.10-1.41)	.001	.001
Model 2	1 [reference]	NA	1.06 (0.96-1.16)	.24	1.15 (1.02-1.31)	.03	.002
Model 3	1 [reference]	NA	1.04 (0.95-1.15)	.36	1.16 (1.02-1.33)	.02	.002
**Artificially sweetened beverages**							
Model 1	1 [reference]	NA	1.23 (1.09-1.39)	.001	1.52 (1.41-1.64)	<.001	<.001
Model 2	1 [reference]	NA	1.09 (0.97-1.24)	.15	1.36 (1.26-1.47)	<.001	<.001
Model 3	1 [reference]	NA	1.10 (0.97-1.24)	.16	1.36 (1.26-1.46)	<.001	<.001
**Natural juices**							
Model 1	1 [reference]	NA	0.85 (0.79-0.91)	<.001	0.88 (0.78-1.01)	.06	<.001
Model 2	1 [reference]	NA	0.91 (0.85–0.97)	.004	0.91 (0.80-1.04)	.17	.007
Model 3	1 [reference]	NA	0.93 (0.87-0.99)	.03	0.95 (0.83-1.08)	.43	.07

Abbreviations: HR, hazard ratio; CI, confidence interval; NA, not applicable.
[a]This analysis included 46 203 participants. Composite chronic kidney disease outcome was defined based on diagnosis codes, or measurements of eGFR <60 mL/min/1.73 m^2, whichever came first. Healthy diet score and models as in the footnote to Table 2

© 2024 Heo GY et al. *JAMA Network Open*.

eTable 5. Risk of incident chronic kidney disease by category of beverage intake with four-way decomposition by sugar intake and BMI

Four-way decomposition	Sugar sweetened beverages			Artificially sweetened beverages		
	Estimates (95% CI)	P value	Proportion (%)	Estimates (95% CI)	P value	Proportion (%)
**By sugar intake**						
Total excess relative risk	0.16 (0.08-0.24)	<.001	-	0.20 (0.13-0.27)	<.001	
Excess relative risk due to direct effect	0.14 (0.01-0.26)	.03	87.5	0.20 (0.13-0.26)	<.001	100.0
Excess relative risk due to reference interaction	0.00 (-0.01-0.01)	.68	0.0	0.00 (-0.00-0.00)	.97	0.0
Excess relative risk due to mediated interaction	-0.01 (-0.07-0.40)	.65	-6.2	0.00 (-0.00-0.00)	.92	0.0
Excess relative risk due to pure indirect effect	0.03 (0.00-0.06)	.04	18.7	0.00 (0.00-0.00)	.02	0.0
Total effect relative risk ratio	1.16 (1.08-1.24)	<.001		1.20 (1.13-1.27)	<.001	
**By BMI**						
Total excess relative risk	0.15 (0.04-0.27)	.008		0.33 (0.25-0.41)	<.001	
Excess relative risk due to direct effect	0.12 (0.01-0.24)	.04	80.0	0.25 (0.16-0.34)	<.001	75.7
Excess relative risk due to reference interaction	0.00 (-0.01-0.01)	.89	0.0	0.00 (-0.01-0.00)	.21	0.0
Excess relative risk due to mediated interaction	0.00 (-0.01-0.01)	.85	0.0	-0.01 (-0.04-0.03)	.79	-3.0
Excess relative risk due to pure indirect effect	0.03 (0.03-0.04)	<.001	20.0	0.09 (0.08-0.11)	<.001	27.3
Total effect relative risk ratio	1.16 (1.04-1.27)	.008		1.33 (1.25-1.41)	<.001	

Note: The estimates were result from mediation analysis by sugar intake and BMI in mean value. The proportion was defined as the ratio of each effect (direct effect, reference interaction, mediated interaction, indirect effect) to the total effect. The model was adjusted for age, sex, ethnic background, Townsend deprivation index, ethnic background, Townsend deprivation index, alcohol consumption status, smoking status, body mass index, and physical activity, comorbidities (hypertension, diabetes, and cardiovascular disease), the use of medications (renin-angiotensin-aldosterone system inhibitor and statins), dietary intake (total energy, total sugar and healthy diet score [a]), and laboratory measurements (estimated glomerular filtration rate, urine albumin to creatinine ratio, low-density lipoprotein cholesterol, and high-sensitive C-reactive protein).

a Healthy diet score was calculated based on seven dietary factors according to recommendations for dietary priorities on cardiometabolic health, ranged from 0 to 7. Each favorable dietary factor contributed one point to the overall score: total vegetables ≥4 servings/day; total fruit ≥ 4 servings/day; total fish ≥2 servings/week; processed meat ≤1 serving/week; red meat ≤1.5 servings/week; whole grains ≥3 servings/day; refined grains ≤1.5 servings/day.

© 2024 Heo GY et al. *JAMA Network Open*.

eTable 6. Risk of incident chronic kidney disease by category of beverage intake from first completed dietary questionnaire

Incident chronic kidney disease[a]	0 serving/day HR (95% CI)	P value	>0-1 servings/day HR (95% CI)	P value	>1 servings/day HR (95% CI)	P value	P for trend
**Sugar sweetened beverages**							
Model 1	1 [reference]	NA	1.13 (1.04-1.23)	.004	1.32 (1.18-1.49)	<.001	<.001
Model 2	1 [reference]	NA	1.10 (1.02-1.20)	.02	1.20 (1.07-1.35)	.003	<.001
Model 3	1 [reference]	NA	1.07 (0.98-1.16)	.14	1.13 (1.01-1.27)	.04	.03
**Artificially sweetened beverages**							
Model 1	1 [reference]	NA	1.31 (1.19-1.45)	<.001	1.65 (1.46-1.87)	<.001	<.001
Model 2	1 [reference]	NA	1.12 (1.01-1.24)	.03	1.22 (1.08-1.38)	.002	<.001
Model 3	1 [reference]	NA	1.12 (1.01-1.23)	.04	1.24 (1.09-1.40)	.001	<.001
**Natural juices**							
Model 1	1 [reference]	NA	0.87 (0.82-0.93)	<.001	0.87 (0.77-1.00)	.05	<.001
Model 2	1 [reference]	NA	0.94 (0.88-1.00)	.06	0.91 (0.79-1.03)	.14	.03
Model 3	1 [reference]	NA	0.95 (0.89-1.02)	.14	0.93 (0.81-1.07)	.31	.11

Abbreviations: HR, hazard ratio; CI, confidence interval; NA, not applicable.
[a]This analysis included 46 203 participants. Healthy diet score and models as in the footnote to Table 2

© 2024 Heo GY et al. *JAMA Network Open*.

eTable 7. Risk of incident chronic kidney disease by category of beverage intake, excluding participants developing events during first 3 years of follow-up

Incident chronic kidney disease[a]	0 serving/day HR (95% CI)	P value	>0-1 servings/day HR (95% CI)	P value	>1 servings/day HR (95% CI)	P value	P for trend
**Sugar sweetened beverages**							
Model 1	1 [reference]	NA	1.11 (1.03-1.21)	.01	1.41 (1.23-1.62)	<.001	<.001
Model 2	1 [reference]	NA	1.10 (1.02-1.20)	.02	1.26 (1.10-1.46)	.001	<.001
Model 3	1 [reference]	NA	1.06 (0.97-1.15)	.20	1.17 (1.01-1.36)	.04	.03
**Artificially sweetened beverages**							
Model 1	1 [reference]	NA	1.34 (1.22-1.48)	<.001	1.76 (1.53-2.04)	<.001	<.001
Model 2	1 [reference]	NA	1.16 (1.05-1.28)	.003	1.26 (1.08-1.45)	.002	<.001
Model 3	1 [reference]	NA	1.16 (1.05-1.28)	.004	1.28 (1.11-1.49)	.001	<.001
**Natural juices**							
Model 1	1 [reference]	NA	0.84 (0.78-0.91)	<.001	0.90 (0.78-1.04)	.15	<.001
Model 2	1 [reference]	NA	0.92 (0.85–0.99)	.01	0.96 (0.83-1.11)	.70	.07
Model 3	1 [reference]	NA	0.92 (0.85-0.99)	.02	0.97 (0.83-1.12)	.65	.09

Abbreviations: HR, hazard ratio; CI, confidence interval; NA, not applicable.
[a]This analysis included 125 554 participants. Healthy diet score and models as in the footnote to Table 2

© 2024 Heo GY et al. *JAMA Network Open*.

eTable 8. Risk of incident chronic kidney disease by category of beverage intake among the participants who conducted two or more dietary assessments

Incident chronic kidney disease[a]	0 serving/day HR (95% CI)	P value	>0-1 servings/day HR (95% CI)	P value	>1 servings/day HR (95% CI)	P value	P for trend
**Sugar sweetened beverages**							
Model 1	1 [reference]	NA	1.15 (1.05-1.25)	.002	1.58 (1.35-1.85)	<.001	<.001
Model 2	1 [reference]	NA	1.12 (1.03-1.22)	.01	1.42 (1.21-1.66)	<.001	<.001
Model 3	1 [reference]	NA	1.06 (0.97-1.16)	.18	1.29 (1.09-1.52)	.003	.006
**Artificially sweetened beverages**							
Model 1	1 [reference]	NA	1.30 (1.17-1.43)	<.001	1.87 (1.60-2.19)	<.001	<.001
Model 2	1 [reference]	NA	1.09 (0.99-1.21)	.08	1.31 (1.11-1.54)	.001	<.001
Model 3	1 [reference]	NA	1.10 (0.99-1.21)	.08	1.35 (1.15-1.59)	<.001	<.001
**Natural juices**							
Model 1	1 [reference]	NA	0.86 (0.79-0.94)	<.001	0.94 (0.80-1.09)	.41	.01
Model 2	1 [reference]	NA	0.93 (0.85–1.01)	.07	1.00 (0.86-1.17)	.95	.32
Model 3	1 [reference]	NA	0.92 (0.85-1.01)	.07	0.98 (0.84-1.15)	.82	.24

Abbreviations: HR, hazard ratio; CI, confidence interval; NA, not applicable.
[a]Total 77 481 participants conducted dietary assessments more than two times. Healthy diet score and models as in the footnote to Table 2

© 2024 Heo GY et al. *JAMA Network Open*.

eTable 9. Risk of incident chronic kidney disease by category of beverage intake by adjustment of household income

Incident chronic kidney disease[a]	0 serving/day HR (95% CI)	P value	>0-1 servings/day HR (95% CI)	P value	>1 servings/day HR (95% CI)	P value	P for trend
**Sugar sweetened beverages**							
Model 1	1 [reference]	NA	1.09 (1.02-1.17)	.01	1.42 (1.26-1.59)	<.001	<.001
Model 2	1 [reference]	NA	1.08 (1.01-1.15)	.03	1.27 (1.14-1.43)	<.001	<.001
Model 3	1 [reference]	NA	1.04 (0.97-1.11)	.28	1.20 (1.06-1.36)	.003	.04
**Artificially sweetened beverages**							
Model 1	1 [reference]	NA	1.09 (1.02-1.17)	.01	1.42 (1.26-1.59)	<.001	<.001
Model 2	1 [reference]	NA	1.10 (1.01-1.20)	.02	1.23 (1.09-1.39)	<.001	<.001
Model 3	1 [reference]	NA	1.10 (1.01-1.20)	.02	1.27 (1.12-1.43)	<.001	.002
**Natural juices**							
Model 1	1 [reference]	NA	0.85 (0.80-0.91)	.001	0.92 (0.82-1.04)	.17	<.001
Model 2	1 [reference]	NA	0.92 (0.87–0.98)	.01	0.98 (0.87-1.10)	.70	.07
Model 3	1 [reference]	NA	0.93 (0.87-0.99)	.002	0.99 (0.87-1.11)	.82	.38

Abbreviations: HR, hazard ratio; CI, confidence interval; NA, not applicable.
[a]This analysis included 117 278 participants with household income data. The model adjusted for household income, rather than Townsend deprivation index. Model 1 adjusted for age, sex; Model 2: Model 1 + ethnic background, income, alcohol consumption status, smoking status, body mass index, and physical activity, comorbidities (hypertension, diabetes, and cardiovascular disease) and the use of medications (renin-angiotensin-aldosterone system inhibitor and statins). Model 3: Model 2 + dietary intake (total energy, total sugar, and healthy diet score), and laboratory measurements (estimated glomerular filtration rate, urine albumin to creatinine ratio, low-density lipoprotein cholesterol, and high-sensitive C-reactive protein). Healthy diet score as in the footnote to Table 2

© 2024 Heo GY et al. *JAMA Network Open*.

eTable 10. Risk of incident chronic kidney disease by category of beverage intake after using median imputation, missing indicator methods and multiple imputation by chain equation

Incident chronic kidney disease[a]	0 serving/day HR (95% CI)	P value	>0-1 servings/day HR (95% CI)	P value	>1 servings/day HR (95% CI)	P value	P for trend
**Sugar sweetened beverages**							
Median imputation	1 [reference]	NA	1.04 (0.97-1.12)	.26	1.19 (1.05-1.34)	.006	.01
Missing indicator methods	1 [reference]	NA	1.11 (1.06-1.16)	<.001	1.19 (1.09-1.30)	<.001	<.001
Multiple imputation by chain equation	1 [reference]	NA	1.06 (0.99-1.13)	.05	1.19 (1.07-1.33)	.002	.001
**Artificially sweetened beverages**							
Median imputation	1 [reference]	NA	1.10 (1.01-1.20)	.02	1.26 (1.12-1.43)	<.001	<.001
Missing indicator methods	1 [reference]	NA	1.12 (1.05-1.17)	<.001	1.27 (1.17-1.38)	<.001	<.001
Multiple imputation by chain equation	1 [reference]	NA	1.10 (1.02-1.19)	.01	1.27 (1.13-1.41)	<.001	<.001
**Natural juices**							
Median imputation	1 [reference]	NA	0.93 (0.87-0.99)	.03	0.99 (0.88-1.12)	0.90	.14
Missing indicator methods	1 [reference]	NA	0.97 (0.93-1.01)	.15	1.00 (0.91-1.09)	0.95	.34
Multiple imputation by chain equation	1 [reference]	NA	0.94 (0.88-0.99)	.03	1.00 (0.89-1.11)	0.95	.15

Abbreviations: HR, hazard ratio; CI, confidence interval; NA, not applicable.
[a]Total 151 400 participants were included in this analysis. Townsend deprivation index, alcohol consumption status, smoking status, body mass index, physical activity, low-density lipoprotein cholesterol, and high-sensitive C-reactive protein were imputed using median imputation, missing indicator methods and multiple imputation by chain equation. Model adjusted for age, sex, ethnic background, Townsend Deprivation Index, alcohol consumption status, smoking status, body mass index, physical activity, comorbidity (hypertension, diabetes, and cardiovascular disease), the use of medication (renin-angiotensin-aldosterone system inhibitor and statins), dietary intake (total energy, total sugar, and healthy diet scoreba), and laboratory measurements (estimated glomerular filtration rate, urine albumin to creatinine ratio, low-density lipoprotein cholesterol, and high-sensitive C-reactive protein). Healthy diet score as in the footnote to Table 2

© 2024 Heo GY et al. *JAMA Network Open*.

eFigure. Multivariable-adjusted hazard ratios for incident chronic kidney disease stratified by subgroup

(A)

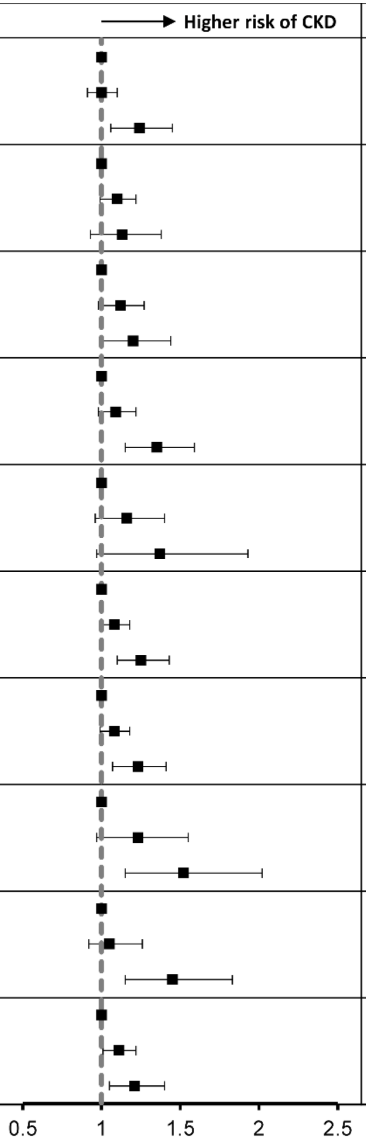

Subgroup	SSB	HR (95% CI)	P for interaction
**Male**	0/day	1.00 (Reference)	.69
	>0-1/day	1.00 (0.91-1.10)	
	>1day	1.24 (1.06-1.45)	
**Female**	0/day	1.00 (Reference)	
	>0-1/day	1.10 (0.99-1.22)	
	>1day	1.13 (0.93-1.38)	
**Age <60 years**	0/day	1.00 (Reference)	.35
	>0-1/day	1.12 (0.98-1.27)	
	>1day	1.20 (1.00-1.44)	
**Age ≥60 years**	0/day	1.00 (Reference)	
	>0-1/day	1.09 (0.98-1.22)	
	>1day	1.35 (1.15-1.59)	
**BMI <25 kg/m^2**	0/day	1.00 (Reference)	.12
	>0-1/day	1.16 (0.96-1.40)	
	>1day	1.37 (0.97-1.93)	
**BMI ≥25 kg/m^2**	0/day	1.00 (Reference)	
	>0-1/day	1.08 (0.99-1.18)	
	>1day	1.25 (1.10-1.43)	
**Diabetes history (-)**	0/day	1.00 (Reference)	.05
	>0-1/day	1.08 (0.99-1.18)	
	>1day	1.23 (1.07-1.41)	
**Diabetes history (+)**	0/day	1.00 (Reference)	
	>0-1/day	1.23 (0.97-1.55)	
	>1day	1.52 (1.15-2.02)	
**hs-CRP <1 mg/L**	0/day	1.00 (Reference)	.61
	>0-1/day	1.05 (0.92-1.26)	
	>1day	1.45 (1.15-1.83)	
**hs-CRP ≥1 mg/L**	0/day	1.00 (Reference)	
	>0-1/day	1.11 (1.01-1.22)	
	>1day	1.21 (1.05-1.40)	

© 2024 Heo GY et al. *JAMA Network Open*.

eFigure (B)

Subgroup	ASB	HR (95% CI)	P for interaction
**Male**	0/day	1.00 (Reference)	.75
	>0-1/day	1.10 (0.98-1.24)	
	>1day	1.24 (1.05-1.47)	
**Female**	0/day	1.00 (Reference)	
	>0-1/day	1.10 (0.98-1.24)	
	>1day	1.28 (1.07-1.52)	
**Age <60 years**	0/day	1.00 (Reference)	.87
	>0-1/day	1.12 (0.97-1.26)	
	>1day	1.18 (0.98-1.42)	
**Age ≥60 years**	0/day	1.00 (Reference)	
	>0-1/day	1.10 (0.98-1.22)	
	>1day	1.34 (1.14-1.58)	
**BMI <25 kg/m²**	0/day	1.00 (Reference)	.36
	>0-1/day	1.16 (0.96-1.39)	
	>1day	1.35 (0.96-1.91)	
**BMI ≥25 kg/m²**	0/day	1.00 (Reference)	
	>0-1/day	1.08 (0.98-1.18)	
	>1day	1.24 (1.09-1.42)	
**Diabetes history (-)**	0/day	1.00 (Reference)	.22
	>0-1/day	1.07 (0.98-1.17)	
	>1day	1.22 (1.06-1.39)	
**Diabetes history (+)**	0/day	1.00 (Reference)	
	>0-1/day	1.29 (1.02-1.63)	
	>1day	1.53 (1.15-2.03)	
**hs-CRP <1 mg/L**	0/day	1.00 (Reference)	.41
	>0-1/day	1.07 (1.07-1.11)	
	>1day	1.43 (1.13-1.80)	
**hs-CRP ≥1 mg/L**	0/day	1.00 (Reference)	
	>0-1/day	1.11 (1.01-1.23)	
	>1day	1.21 (1.04-1.39)	

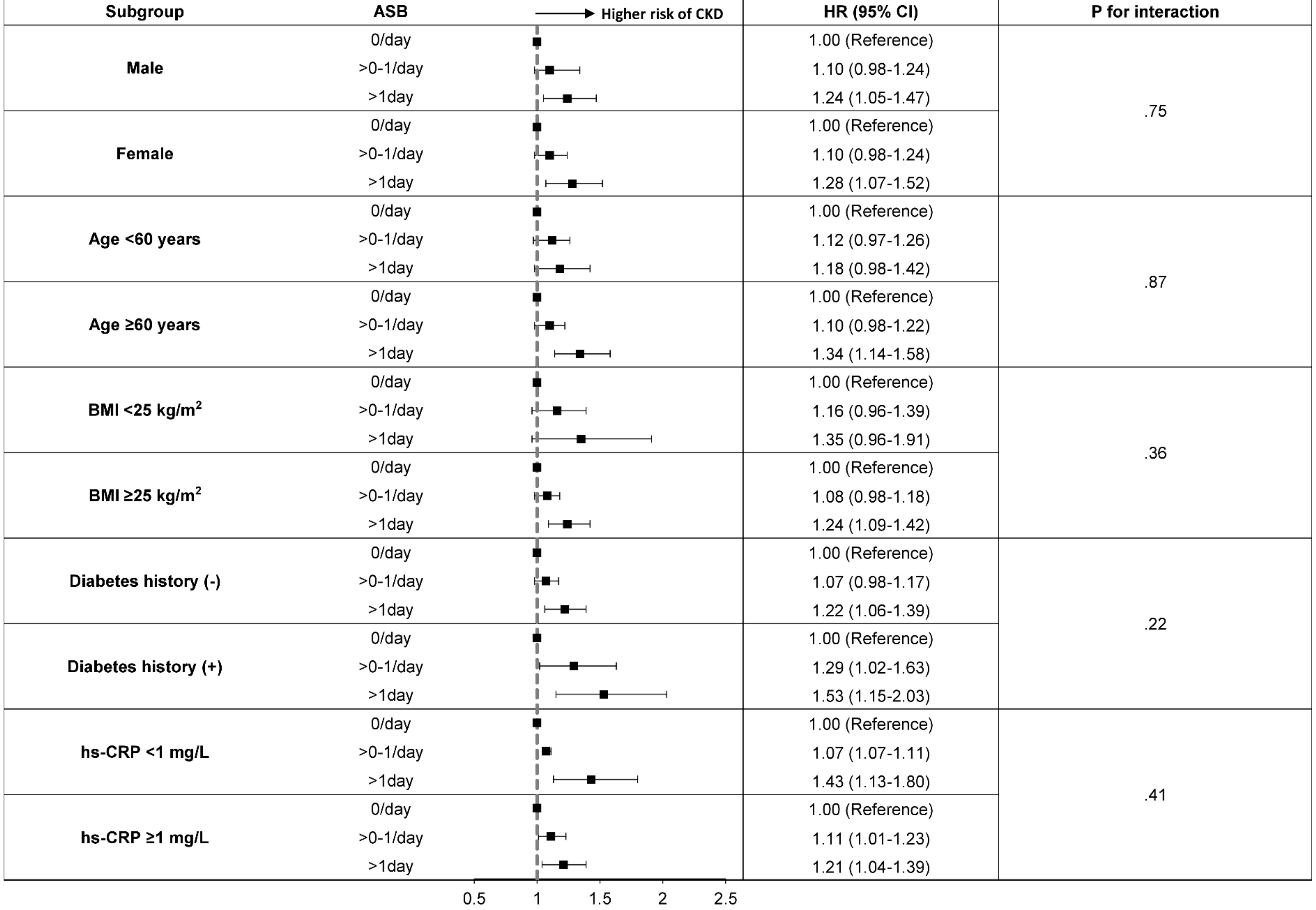

© 2024 Heo GY et al. *JAMA Network Open*.

eFigure (C)

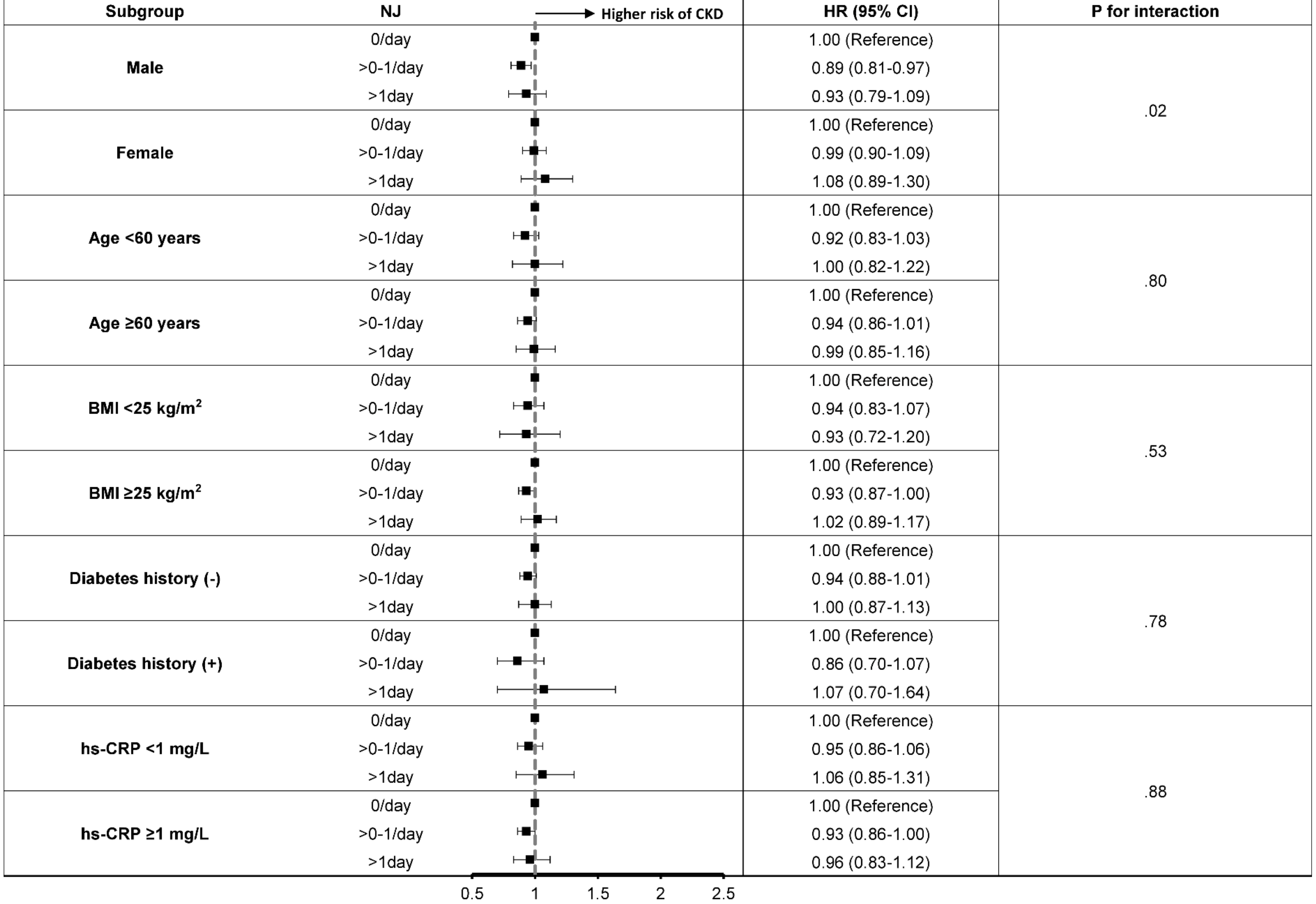

Subgroup	NJ	HR (95% CI)	P for interaction
**Male**	0/day	1.00 (Reference)	.02
	>0-1/day	0.89 (0.81-0.97)	
	>1day	0.93 (0.79-1.09)	
**Female**	0/day	1.00 (Reference)	
	>0-1/day	0.99 (0.90-1.09)	
	>1day	1.08 (0.89-1.30)	
**Age <60 years**	0/day	1.00 (Reference)	.80
	>0-1/day	0.92 (0.83-1.03)	
	>1day	1.00 (0.82-1.22)	
**Age ≥60 years**	0/day	1.00 (Reference)	
	>0-1/day	0.94 (0.86-1.01)	
	>1day	0.99 (0.85-1.16)	
**BMI <25 kg/m^2**	0/day	1.00 (Reference)	.53
	>0-1/day	0.94 (0.83-1.07)	
	>1day	0.93 (0.72-1.20)	
**BMI ≥25 kg/m^2**	0/day	1.00 (Reference)	
	>0-1/day	0.93 (0.87-1.00)	
	>1day	1.02 (0.89-1.17)	
**Diabetes history (-)**	0/day	1.00 (Reference)	.78
	>0-1/day	0.94 (0.88-1.01)	
	>1day	1.00 (0.87-1.13)	
**Diabetes history (+)**	0/day	1.00 (Reference)	
	>0-1/day	0.86 (0.70-1.07)	
	>1day	1.07 (0.70-1.64)	
**hs-CRP <1 mg/L**	0/day	1.00 (Reference)	.88
	>0-1/day	0.95 (0.86-1.06)	
	>1day	1.06 (0.85-1.31)	
**hs-CRP ≥1 mg/L**	0/day	1.00 (Reference)	
	>0-1/day	0.93 (0.86-1.00)	
	>1day	0.96 (0.83-1.12)	

According to three categories of A) sugar-sweetened beverage, B) artificially sweetened beverage and C) natural juice., the HRs were adjusted for age, sex, ethnic background, Townsend deprivation index, ethnic background, Townsend deprivation index, alcohol consumption status, smoking status, BMI, physical activity, comorbidities (hypertension, diabetes, and cardiovascular disease), the use of medications (renin-angiotensin-aldosterone system inhibitor and statins), dietary intake (total energy, total sugar, and healthy diet score[a]), and laboratory measurements (estimated glomerular filtration rate, urine albumin to creatinine ratio, low-density lipoprotein cholesterol, and hs-CRP).

© 2024 Heo GY et al. *JAMA Network Open*.

[a] Healthy diet score was calculated based on seven dietary factors according to recommendations for dietary priorities on cardiometabolic health, ranged from 0 to 7. Each favorable dietary factor contributed one point to the overall score: total vegetables ≥4 servings/day; total fruit ≥ 4 servings/day; total fish ≥2 servings/week; processed meat ≤1 serving/week; red meat ≤1.5 servings/week; whole grains ≥3 servings/day; refined grains ≤1.5 servings/day.

Abbreviations: HR, hazard ratio; CI, confidence intervals; BMI, body mass index; hs-CRP, high-sensitive C-reactive protein

© 2024 Heo GY et al. *JAMA Network Open*.
