# Supplementary material for: Sweetened Beverage Intake and Incident Chronic Kidney Disease in the UK Biobank Study
Source: JAMA Netw Open. 2024 Feb 28;7(2):e2356885. doi: 10.1001/jamanetworkopen.2023.56885 (PMC10902724; doi:10.1001/jamanetworkopen.2023.56885)
Supplement: Supplement 2. — Data Sharing Statement [file jamanetwopen-e2356885-s002.pdf]

Data Sharing Statement

Heo. Sweetened Beverage Intake and Incident Chronic Kidney Disease in the UK Biobank Study. *JAMA Netw Open*. Published February 28, 2024.
doi:10.1001/jamanetworkopen.2023.56885

Data
Data available: No

Additional Information
Explanation for why data not available: Researchers can use UK Biobank resources to access the data used in this study. UK Biobank Data will be made available on the UK Biobank Consortium website.
